# Development of the Hearing Dog Evaluation and Reporting Overview (HERO): A Novel Measure for Hearing Dog Welfare

**DOI:** 10.3390/ani15060785

**Published:** 2025-03-10

**Authors:** Charis Koh, Holly Chillingworth, Carlie Driscoll, Jessica Hill, Edward Narayan

**Affiliations:** 1 School of Health & Rehabilitation Sciences, University of Queensland, Brisbane 4072, Australia; c.koh@uq.net.au (C.K.);; 2 School of Agriculture & Food Science, University of Queensland, Brisbane 4072, Australia; 3 Faculty of Science and Engineering, Southern Cross University, Lismore 2480, Australia

**Keywords:** assistance animals, five domains, hearing dogs, welfare, wellbeing

## Abstract

This paper delves into the development and implications of the Hearing Dog Evaluation and Reporting Overview (HERO) tool, which is a novel initiative built on the Five Domains animal welfare model to comprehensively assess the wellbeing of Hearing Dogs. The HERO’s creation involved two phases. In Phase One, an expert panel was surveyed using an electronic Delphi technique to gather consensus on items for the tool. This phase culminated in 28 items, exhaustively covered the Five Domains, and offered a holistic evaluation of Hearing Dog welfare. Phase One complemented existing assistance animal standards and emphasised the importance of a quality environment and continuous wellbeing monitoring. Phase Two involved 23 Hearing Dog owners, to assess the tool’s usability, particularly its user-friendliness and perceived value. Concerns about handlers’ ability to identify welfare indicators were raised, highlighting the need for additional development of resources and guidance. This preliminary study has shown that the HERO is a promising tool for understanding and promoting Hearing Dog welfare. Future research will look to validate the tool by drawing from expert observations and physiological measures, tailoring survey questions, and creating a user-sharing platform.

## Simple Summary

Hearing Dogs provide an invaluable source of support and companionship for individuals with hearing loss. These specially trained assistance animals serve as alert systems, enabling their owners to navigate the world more independently and securely. Although the positive impacts of Hearing Dogs on the lives of their owners are well-documented, there has been a notable gap in research regarding the welfare of these service animals. In particular, a systematic welfare diagnostic for Hearing Dogs is needed to influence best practises and inform care decisions. This research explored the development and implications of the Hearing Dog Evaluation and Reporting Overview Tool (HERO), a novel welfare measure rooted in the Five Domains model. The study involved two phases: Phase One employed an expert panel to determine the tool’s items, whilst Phase Two piloted the tool with Hearing Dog owners, investigating its useability and relevance. The 28-item tool, following further refinement, has the potential to establish benchmarks, track wellbeing changes over time, contribute to evidence-based practises, and inform policymaking in the field of assistance animals.

## 1. Introduction

Hearing Dogs assist those with hearing loss in their daily living. Hearing Dogs are a type of assistance animal who work to enhance their owner’s/handler’s independence and safety by alerting them to important environmental sounds (e.g., doorbells, telephone ringing, fire alarms, warning sounds, and sirens in public areas). Studies have shown that they can significantly improve quality of life by enhancing mental health, increasing active living, and elevating feelings of safety and independence [1,2,3,4,5,6]. Since 1982, the primary source of Hearing Dogs within Australia, the Australian Lions Hearing Dog Programme, has provided over 650 dogs to owners, and aims to have 1000 dogs in service at any one time [7].

While Hearing Dogs are known to improve the quality of life of their handlers, the welfare status of the dogs themselves is not well understood. As stated by Townsend and Gee [8] (p. 13), “fulfilling our responsibility to our canine co-workers requires a stance that their welfare stands on equal footing with that of our clients”. Furthermore, mutual benefit must be demonstrable for all humans and animals involved in animal-assisted interventions or services. Currently, the welfare of Hearing Dogs is lacking in research and there is no tool to operationalise welfare standards.

Emerging studies of assistance and therapy dogs can be used to infer challenges that Hearing Dogs may face. Such examples may include high daily workload; limited daily human–animal interaction; insufficient canine–canine socialising; inhospitable transportation environments; insufficient quality of home environment, food and water; and lack of downtime [9,10]. A study of canine stress during animal-assisted therapy found that dogs are susceptible to stress associated with environmental, physical, and social challenges, which can lead to the deterioration of physical, social, emotional, and mental health [8]. Higher states of arousal (e.g., salivary cortisol levels, observable behaviours, heart rate, respiratory rate, salivary oxytocin, tympanic membrane temperature, and observed stress levels) were also found when therapy dogs worked in an unfamiliar environment [11].

As research in animal welfare progresses, it has become evident that welfare cannot be objectively observed, but rather is the qualia of an animal that requires careful assessment and consideration to properly define [12]. Dogs are not merely tools but are sentient beings who experience highly nuanced emotions and should be treated as such [8]. A review of the literature highlights that limited standards have been established to inform the correct treatment of Hearing Dogs, notably within legislation, Hearing Dog programmes, and among Hearing Dog providers. Within Australia, the following standards are available for assistance animals:

1. The Animal Therapies Limited (ATL) Code of Conduct for the Animal-Assisted Services Sector [13]. This code of conduct details best practises for the care and welfare of assistance animals (including but not limited to Hearing Dogs). Specific standards include that handlers have the following: a general knowledge of animal welfare (B2.5); can provide adequate housing, nutrition, transportation, veterinary care, exercise, environmental enrichment, rest and recreation, and companionship (B2.6); are able to monitor for signs of stress, discomfort, fear, illness, injury and ageing (B2.10); and ensure the animal is well groomed (B2.13) and free of parasites (B2.15), among others. In particular, the code states that assistance animals should be viewed as sentient beings whose wellbeing, including the right to flourish, should be of paramount concern (B2.6).
2. The Assistance Dogs International (ADI) accreditation standards [14]. These standards state that for an assistance dog training programme to gain accreditation, they must demonstrate the following: appropriate healthcare management and an ongoing plan; provision of humane care toward all dogs; commitment to working with veterinarians; commitment to upholding the dog’s welfare for the lifetime of the dog; and, appropriate and safe kennelling and shelter that upholds the needs of the dog. Unlike the ATL Code of Conduct, the ADI standards do not specify a dog’s need to flourish. They also do not specify ongoing surveillance of assistance dogs and their owners past the period of gaining accreditation.

As interest in and awareness of animal wellbeing grows within the general public [15], it becomes increasingly important that welfare standards are operationalised and upheld. The usefulness of a welfare assessment tool cannot be overstated, as it can serve as a powerful evidence-based standard to systematically evaluate animal quality of life and wellbeing. Such a tool can provide valuable insight for stakeholders (e.g., Hearing Dog owners, researchers, and animal care professionals), by enabling measurement and monitoring of various indicators of welfare, which can guide care behaviour through targeted interventions that focus on highlighted areas of concern. A reliable tool will maximise understanding of Hearing Dog wellbeing and support data-driven decisions [16]. It could also facilitate benchmark-setting, progress-tracking, and inform judgement-making, thereby contributing to evidence-based practises and policy development [17,18].

Originally, the gold standard for evaluating animal welfare was the Five Freedoms model, which had a wide uptake due to its clear theoretical distinction of five welfare factors and its ability to help mitigate negative animal welfare experiences [19]. However, its measurements were subjective and there was no standardised method of quantification, and its focus on mitigation of negative welfare factors did not provide a pathway to enhance positive welfare experiences of the animal [20,21].

The Five Domains, developed by Prof. David Mellor and Dr. Cam Reid in 1994, is a model of animal welfare that promotes best practises for positive welfare outcomes and employs an observational method that infers the likelihood of a positive or negative welfare condition [12]. With foundations in the Five Freedoms, Mellor’s model reflected a shift in the field of animal welfare from simply considering an animal’s basic needs, to a holistic understanding of its emotions and affective states [20]. A focus was placed on positive welfare factors and the facilitation of the animal’s ability to flourish. It has been widely considered that merely mitigating negative experiences in an animal’s life and providing for survival-critical needs only will lead to a neutral welfare state at best, neither particularly bad or good, whereas introducing and providing positive welfare experiences leads to positive welfare outcomes and ultimately a life worth living [12,22,23].

The model employs five categories: (1) nutrition, (2) environment, (3) health, (4) behavioural interactions, and (5) mental state, and has been regularly updated to encompass new developments in animal welfare, science, and philosophy [24]. The first four domains are observable and measurable factors (e.g., amount and quality of food and water, physical health), with integration of these factors painting a picture of mental experiences and affective states of the animal. Research suggests that the mental state of animals is closely linked to their welfare state, which allows us to indirectly observe the fifth domain through observations of domains 1 to 4 [12,25,26].

In this study, we aimed to develop a practical prototype welfare assessment tool for Hearing Dogs in line with the Five Domains [26] and to conduct a preliminary assessment of its usability. The usability of an assessment tool for the evaluation and promotion of animal wellbeing is a crucial element for effective implementation and widespread adoption. A well-designed and user-friendly assessment tool enhances user performance and satisfaction by facilitating efficient navigation via an intuitive interface and clear instructions [27,28]. Optimisation of the assessment tool can reduce respondent burden, eliminate confusion, minimise participant attrition, and increase the likelihood of correctly identifying areas of concern [16].

## 2. Materials and Methods

This prospective study comprised two phases: Phase One featured the initial development of the questionnaire, while Phase Two focused on evaluating the usability and usefulness of the assessment tool. Ethical clearance was obtained from the University of Queensland Health and Behavioural Science Ethics Committee, Brisbane, Australia (Ethics ID numbers 2022/HE001141 and 2023/HE000439).

### 2.1. Phase One

#### 2.1.1. Participants

Subject experts for participation in a Delphi panel were purposively identified via key professional associations and networks in Australia; they were invited to voluntarily participate directly via email. A total of nine invitations were sent, with a response rate of 77.8% (*n* = 7). Informed consent was obtained, and all participants remained anonymous. Participants were selected based on their qualifications that collectively accounted for the following four categories: (1) expertise in the field of animal welfare, (2) expertise in the field of Hearing Dogs, (3) Hearing Dog trainer or handler, or (4) expertise in the field of assistance and therapy animals. Of the seven experts that participated in the study, six (86%) had a PhD in their respective fields of biology, animal welfare, or agriculture and animal sciences, and they were either a senior fellow, senior lecturer, research associate, or clinical researcher with an Australian university. Two (29%) of the experts had close affiliations with Hearing Dog or therapy animal welfare and training bodies. Three (43%) were practising or research veterinarians, while two (29%) were assistance dog trainers.

#### 2.1.2. Procedure

The formation of a welfare assessment tool requires a coherent foundation and a uniform, final opinion agreed upon by various experts. Where evidence is insufficient or controversial, a modified Delphi technique is suitable for combining scientific evidence and the literature review with expert opinions, to form guidelines and tools [29,30]. The Delphi technique facilitates systematic convergence of differing opinions until a consensus is reached via the combination of multiple expert opinions and reflections [30,31]. It has proven effective in needs assessment and is often used in healthcare settings to determine practice guidelines and assessment tool development [32]. The classic Delphi technique involves administering open-ended questions to participants to gain their opinions on a particular topic. In subsequent rounds, the results are collated and provided to participants, and after an evaluation of all other opinions, participants are prompted to modify their opinions until a consensus is reached [33]. A modified electronic Delphi (eDelphi) technique was used across three rounds in the current study, to obtain a consensus from a panel of experts on items to be included in a welfare assessment tool for Hearing Dogs.

For round one, participants responded to a questionnaire that was formulated based on the Five Domains [24] and contained five open-ended questions, specifically regarding (1) nutrition, (2) environment, (3) health, (4) behavioural interactions, and (5) mental state, with additional space provided for open-ended feedback and suggestions. The questionnaire was distributed via email and the first tool prototype was developed based on resulting themes. Thematic analysis was performed by author HC, which involved creating a code, identifying themes, and collating supporting comments for each theme, as well as unifying terminology, as suggested by Okoli and Pawlowski [34]. Identified themes were reviewed by the authors CD and JH for agreement. From this analysis, a series of 38 potential items to be included on a Hearing Dog welfare assessment tool were developed by the research team.

For round two, the first prototype items were emailed to the same experts via Qualtrics. The participants were asked to indicate their level of agreement using a 5-point Likert scale, with scores of (1) not at all important, (2) slightly important, (3) moderately important, (4) very important, and (5) extremely important, with the intent to determine consensus on which items should be included in the second prototype of the Hearing Dog welfare assessment tool. Consensus on items was deemed acceptable at 75% agreement (i.e., a score of ‘4’ or ‘5’) among all participants [33,35]. Space for open-ended feedback was provided to further refine items based on expert opinion.

Where an item did not reach consensus in round two, it was either altered or omitted for round three, and any item changes were presented in a different colour. The round three prototype, emailed to experts via Qualitrics, also showed participants the Likert response distributions from the previous round, to allow reflection and the opportunity to change an item answer based on other participants’ responses. Participants were tasked to rate the questions in a similar fashion to round two, resulting in the final Hearing Dog Evaluation and Reporting Overview (HERO) prototype. All electronic data were transferred to Microsoft Excel (Version 2018) after each round for analysis.

### 2.2. Phase Two

#### 2.2.1. Participants

In the second phase, the Australian Lions Hearing Dogs was consulted. Upon request, the CEO of the organisation distributed a Qualtrics survey link—this included a project information sheet and the HERO prototype—to all clients with Hearing Dogs (~200 owners). Participation was voluntary with informed consent and all participants remained anonymous. The survey link was made available for one month and a reminder sent one week before the closure of the study. A total of 25 participants indicated interest to take part in the study—2 dropped out after the first three questions and the remaining 23 participants saw the study to completion.

#### 2.2.2. Procedure

In Phase Two of the study, we delivered the HERO prototype survey devised in Phase One to participants via Qualtrics. The survey consisted of two main components. The first section comprised the 28 items developed in Phase One, with links to additional, informative educational material, selected based on usefulness and consistency with the prior literature. In the second section, participants were asked to rate components of usability of the HERO, including ease of use, clarity, accessibility, and the usefulness of the tool. These seven questions required a Likert-style response and were adapted from Rahman and Rahman [36]. Three additional open-ended questions were also presented for completion.

## 3. Results

### 3.1. Phase One

**Figure 1:**
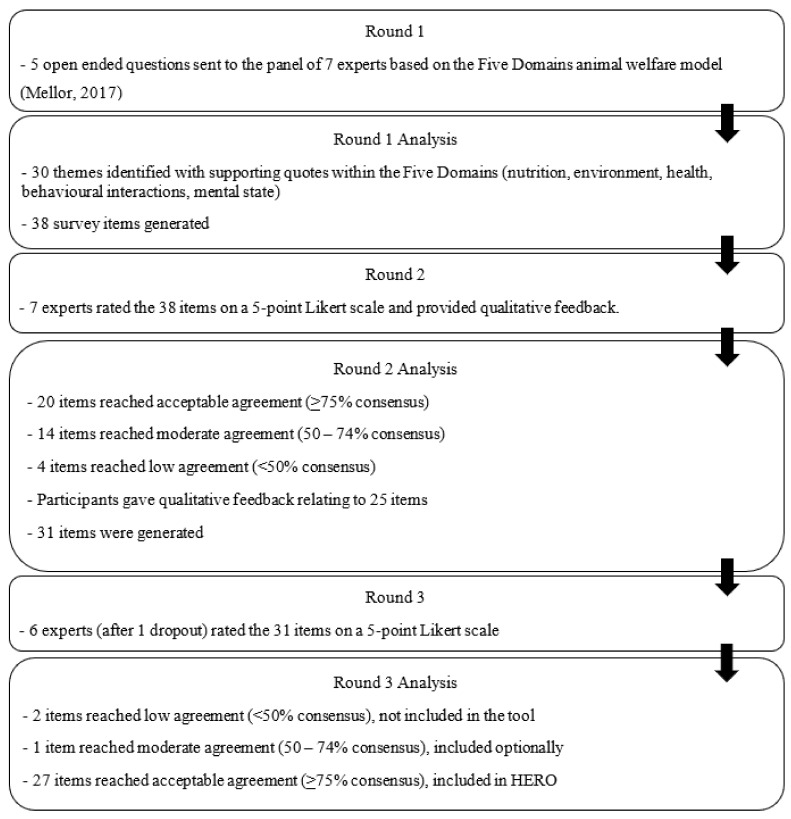
Flowchart of the electronic Delphi study for prototype tool development based on Mellor [26].

The following sections present results from the three-round eDelphi study. This process has been visually represented in Figure 1.

#### 3.1.1. Round One

Experts responded with ideas, concepts, and opinions pertaining to the Five Domains [24]. A thematic analysis [2,34,37] was performed in line with the Five Domains and a total of 30 themes were identified within each of these (i.e., within nutrition, environment, health, behavioural interactions, mental state) (see Table 1). The most common themes were ‘type of food’, ‘quality of environment’, and ‘safety’, which emerged in all the participants’ answers (*n* = 7). These were closely followed by ‘access to water’, ‘ability to withdraw’, ‘monitoring behaviour’, and ‘knowledge of behaviour’ (*n* = 6). The least common themes were ‘dental chews’, ‘vet checks to check for food allergies’, ‘changes in the sound environment’, and ‘use of home remedies’, identified in only one participant’s answers but still considered in the HERO prototype. The number of themes identified within each of the Five Domains was as follows: nutrition (*n* = 7), environment (*n* = 6), health (*n* = 7), behavioural interactions (*n* = 6), and mental state (*n* = 4). One to two possible items were generated by the researchers for each theme, to be included in the prototype of the Hearing Dog welfare assessment tool, producing 38 possible items (see Table 2).

#### 3.1.2. Round Two

Of the 38 items, 20 items reached at least 75% consensus, 14 items obtained between 50% and 74% consensus, and four reached less than 50% consensus (see Table 2). Of the 20 items that reached consensus, 12 were left unchanged in the next version of the tool, 7 items were modified based on participant feedback, and the remaining 1 item was removed due to feedback regarding its similarity to another question.

Alterations were also made to items that reached below 75% agreement (see Table 3). Two items were left unchanged for round three, and six items were modified based on participant feedback. Participants gave 34 pieces of feedback related to 25 items. Of these, 19 were related to item content, and 15 were related to the item wording. Of these 25 items that attracted feedback, 16 were modified, 4 items were merged with another question, 4 items were omitted, and 1 additional question about body condition score was added. Each of the four items that were omitted attracted feedback stating that the topic was covered more thoroughly in another item and was not needed. A total of 31 items were carried to round three.

#### 3.1.3. Round Three

Of the 31 items, a total of 28 items reached consensus with an 86% response rate (*n* = 6) (see Table 4) and 3 items were removed for various reasons (see Table 5). Each of the 19 brought-forward items that reached agreement in round two also reached consensus in round three, regardless of modification, which is indicative of between-round reliability in responses. An additional eight items also reached consensus after modifications. Participants gave feedback such as “This section is much improved from last version”, “the content is good” and “I like the specificity of this question”, indicating improvements in wording and useability. The highest-rated items were “Does your dog have access to fresh, clean water whenever they need it?”, “Is your dog’s place of living comfortable, protected from the weather and not too hot or cold?”, “Is your dog’s place of living safe? This includes an escape-proof home and yard with no hazards.”, and “Does your dog have a space to retreat and withdraw to whenever they need downtime and rest?”.

Participant feedback was provided on 15 of the 31 questions. Five of these feedback items were related to the content of the question, and ten were related to the wording and structure of the question. Considering item consensus and that open-ended feedback was not contradictory to consensus, a pilot HERO tool was created with 28 final items.

### 3.2. Phase Two

This section presents results from the pilot trial of the tool completed by 23 respondents (Hearing Dog owners). Table 6 shows the response type and frequency (i.e., Yes [Y], No [N], and Unsure [U]) for each item. Total scores were very high, with most participants responding ‘Yes’ for most questions, and only one to two people responding ‘No’ or ‘Unsure’ for 10 items.

Subsequently, participants were asked about their opinions of the assessment tool. Quantitative and qualitative analysis was conducted on the responses, and the results are presented in Table 7 and Table 8, respectively. Participant feedback was assessed to evaluate the usability and perceived relevance of HERO.

In terms of usability, participants largely found the assessment tool to be user-friendly. They reported that it was easy to understand, quick to use, with clear wording and easily comprehensible pictures. Additionally, the provided resources were deemed informative and accessible. Participants expressed that the assessment tool was valuable both in understanding their dogs’ needs and in identifying areas for improvement. Questions related to the clarity and ease of use of the assessment tool received more consistent responses with lower standard deviations and variances. On the other hand, questions about the value of the tool in understanding dog needs and assessing improvements, whilst still positive, had more variable responses, as reflected by higher standard deviations and variances.

Participants’ open-ended feedback on their survey experience revealed several important themes. The most common themes included the interest and relevance of the information presented in the survey, with seven participants finding the side information and websites informative, and two participants emphasising that their existing knowledge was reinforced. Two participants noted that the survey did not lead to any change in their behaviours or practises towards their Hearing Dogs. One participant found the survey questions to be common-sense dog owner questions, suggesting that they may not have been tailored specifically to Hearing Dog owners. Three participants also shared some unrelated comments on Hearing Dogs benefits. Three participants offered suggestions for improvement, such as the inclusion of Australian Sign Language (Auslan), reminders about heat and ground conditions for dogs, and providing Hearing Dog information as a resource bundle. Overall, participants generally found the survey to be user-friendly and informative, providing valuable insights into their experiences with their Hearing Dogs. However, some participants indicated a desire for more tailored and specific questions related to Hearing Dogs’ unique needs and experiences.

## 4. Discussion

The development of HERO, guided by the Five Domains [24], represents a step in the right direction in evaluating and promoting the welfare of assistance dogs. The development of the assessment tool involved a meticulous and iterative process. The initial eDelphi survey collected input on five key domains—nutrition, environment, health, behavioural interactions, and mental state—in the welfare assessment of Hearing Dogs [9,12,22,24,38,39]. By focusing on these domains, the study ensured that the essential aspects of Hearing Dog welfare could be evaluated and addressed comprehensively.

The second round of surveying aimed to enhance the tool, based on participant feedback. Among the initial 38 items generated, 20 secured an acceptable consensus rate (i.e., ≥75%). These items were deemed fundamental for HERO, while the remaining 18 underwent further adjustments or were omitted. Notably, items achieving consensus in round two consistently maintained their consensus in round three, demonstrating the tool’s reliability across rounds. In the third round, 28 items (across the five domains) reached an acceptable consensus, demonstrating exhaustive domain coverage. The fact that these items reached a consensus among experts indicates their significance and relevance to the welfare assessment process. The highest-rated items, such as access to clean water, a comfortable living environment, safety, and the provision of a retreat space, align closely with established standards [13,14] and literature [8,10,11], emphasising their importance in supporting the welfare of assistance dogs. This alignment with established practises and research suggests that the developed assessment tool is based on practical foundations. The study also identified items with limited consensus, including questions related to the timing of feeding, supplements, and dental hygiene. These items had less presence in the existing literature concerning assistance dogs.

This study also assessed the usability of the tool and harnessed valuable feedback from participants. In general, Hearing Dog owners found the tool to be user-friendly, particularly appreciating its clarity and ease of use. Nevertheless, there were mixed opinions about the tool’s ability to raise awareness among Hearing Dog owners, to understand their dog’s needs, and to promote welfare improvements. Thematic analysis of participant feedback unveiled several noteworthy themes. Hearing Dog users expressed appreciation for the informative supplementary materials and websites provided in the survey. Some reported that the survey reinforced their existing knowledge, while others expressed an intention to explore further information. A subset of participants observed that some survey questions might be perceived as common-sense inquiries for dog owners. They shared their positive experiences, emphasising the profound impact these dogs had on their lives, particularly in terms of safety and responsiveness to alarms and other auditory cues. Some participants proposed enhancements, such as the inclusion of Australian Sign Language (Auslan) and reminders about weather conditions.

### 4.1. Limitations

One limitation of the study was the use of a small sample of experts, as it may not be representative of the broader expert population. Okoli and Pawlowski [34] noted that a minimum of 10–18 experts is typically considered ideal in the literature. While the current panel’s experts were of diverse backgrounds and were thematically appropriate, expanding the participant pool of experts in future phases could provide a deeper insight into Hearing Dog welfare. Most experts were motivated to ensure quick turnarounds; however, there was one dropout (14%) in the third round. Keeney et al. [30] noted that some dropout is to be expected between Delphi rounds. This did not affect the validity of the study but did affect sample size and increased the chance of a premature consensus, for instance, only five participants were needed to answer four or five on the Likert scale to reach a consensus on an item in round three, as opposed to six participants in round two.

Another limitation was the use of a categorical response scale for the welfare tool, which is in line with concerns raised by some participants. Such a response scale may not allow Hearing Dog owners to sensitively track their progress and improvements over time in the care for their dogs. Amending this tool to adopt a continuous scale with a wider limit could address this issue.

### 4.2. Future Directions

Future research endeavours might explore the practical applications of the tool and its capacity to inform the care and welfare of Hearing Dogs. Further testing can be performed to determine the construct validity of HERO based on comparisons against codified behavioural observations and/or physiological measures that are indicative of wellbeing (e.g., salivary cortisol, tympanic membrane temperature, blood oxygen levels, and preference and aversion tests) [11,40]. Such a development step is particularly important given known limitations in owners’ ability to objectively report on their dogs’ welfare [41]. In an example provided by Malkani et al. [41], it was noted that owners tend to underestimate their dog’s body condition despite the use of a standardised scale [42]. Furthermore, these researchers recommended combining a structured owner report, clinical veterinary examination, veterinary observation of behaviour, and discussion of the animal in question to obtain a more accurate insight into a dog’s welfare, rather than the use of an owner survey tool alone.

In addition, the HERO tool has yet to be tested for reliability, specifically tests of inter-rater, test–retest, and internal consistency. Establishment of the tool’s reliability and validity will provide integral insights into its practical utility. Tailoring survey questions further to cater specifically to the unique needs of Hearing Dogs could also enhance its effectiveness. Additionally, it may be beneficial for welfare to be assessed on a continuous scale for users to capture a broader range of experiences and opinions. The research team could also collect and collate a more extensive resource bundle for users to easily reference and share information regarding the welfare of their Hearing Dogs. Furthermore, it may prove useful to develop an online platform for users to compare their results to population averages, and to share community knowledge, resources, and experiences with other Hearing Dogs owners.

## 5. Conclusions

The creation of a welfare assessment tool, informed by the Five Domains model and subject expert consultation, is necessary to address gaps in the literature surrounding Hearing Dog welfare and to implement practical ways to operationalise best practice standards. This study introduced the HERO tool, which includes key items that should be considered when assessing the welfare of Hearing Dogs. The application of a refined version of the tool may promote awareness regarding the welfare of Hearing Dogs, increase handler uptake, and bolster social and stakeholder support for these assistance animals.

## Figures and Tables

**Table 1:** Round One thematic analysis results (*n* = 7 participants).

Domain	Theme (*n*)	Supporting Quote
Nutrition	Access to water (6)	“Multiple opportunities and sources to access water”
	Nutritional quality of food (7)	“Meeting the nutritional needs of the dog based on breed, age, sex and physical activity”
	Quantity of food (5)	“Quantity of food should be right—to maintain optimum weight.”
	Timing of feeding (4)	“Providing the dog with routine is ideal so that they know when to expect meals. If this is not possible, the dog should be trained to accept food at any time of day.”
	Supplements (2)	“Vitamins and supplements are important.”
	Dental chews (1)	“Dogs should be given chews to aid in dental health—bones are discouraged.”
	Vet checks to identify food allergies (1)	“Routine health checks to check for food allergies that may affect the dog.”
Environment	Quality of environment (7)	“The ability to have shelter and temperature-controlled environments while at home is required to maintain the highest level of standard for the dog.”
	Ability to withdraw (6)	“Provision of a time-out space to allow dog to disengage/rest/recover.”
	Amount of space (4)	“Sufficient space to move around comfortably.”
	Safety (7)	“Provision for safety and protection from extreme weather/situations.”
	Places outside the home (3)	“Bedding and a safe place should be provided when not at home if there for long periods of time.”
	Sound (1)	“Physical environmental changes and subtle changes such as varying frequencies.”
Health	Vet consultation (5)	“Dogs should have an annual veterinary visit at which time they should receive any booster vaccinations, heartworm prevention and get a complete health check including of teeth, respiratory and circulatory systems, eyes and ears.”
	Home remedies (1)	“No home remedies should be used first.”
	Grooming (5)	“Grooming is required under PAT [Public Access Test] and should be closely monitored.”
	Exercise (3)	“Fitness should be maintained with at least daily walks and play time.”
	Deworming (2)	“A worm and parasite program recommended by a local veterinarian should be maintained—this will vary in different regions of Australia.”
	Dental health (1)	“Dental health may be a poor indicator as poor dental health can be attributed to specific breeds.”
	Working age (3)	“Attention should be paid to declining health when dog ages—also dependent on the breed as to what age.”
Behavioural interaction	Monitoring (6)	“Evaluate general behavioural disposition, which is the dog’s general fear, anxiety, arousal, friendliness and sociability. This will give an understanding of how they are in overall situations. Then this needs to be classified into how this disposition changes under different tests and situations like unfamiliar environments, people, animals, etc. This leads to identification of emotional and behavioural thresholds and therefore triggers associated with stress.”
	Knowledge of behaviour (6)	“Handlers should be educated on behaviours related to anxiety (lip licking, yawning, lowered body) to identify if there is a problem with the dog and remove them from that situation.”
	Reason for behavioural issue (2)	“Health problem? Seek vet. Behavioural problem? Seek Trainer.”
	Human interaction (4)	“Dogs should feel comfortable interacting with humans but should not be distracted by them when working.”
	Animal interaction (2)	“Conspecific interaction to facilitate natural need.”
	Positive behaviour experiences (1)	“As much as possible dogs should be given the opportunity for positive behavioural experiences (food enrichment toys, walking, sniffing, playing).”
Mental state	Assessment of mental state (5)	“Assessment for anxiety, stress, fear, frustration would be my top important factors.”
	Contributors to positive mental state (3)	“Extensive socialisation and training and plenty of downtime.”
	Negative mental state (2)	“Signs of a negative mental state (e.g., fearfulness or anxiety) need to be noticed and then the factors leading to them avoided or changed. If a dog becomes too anxious it may need to be retired or not selected for the program. Signs of depression (dog not wanting to go for a walk or not displaying diversity of behaviours or change in behaviour) should also be noted as some people will see these dogs as being very compliant.”
	Consent to engage (2)	“As much as possible choice tests should be given to dogs to ensure they do want to do the work or interact, e.g., pat tests to see if dog actually likes to be petted.”

**Table 2:** Round two items and agreement using a 5-point Likert scale (n = 7), where 1 = not at all important, 2 = somewhat not important, 3 = neutral, 4 = somewhat important, and 5 = extremely important.

Round Two Prototype Item		1-Point (%)	2-Point (%)	3-Point (%)	4-Point (%)	5-Point (%)	Mdn
1	Does your dog have access to fresh, clean water whenever they need it? *	0 (0)	0 (0)	0 (0)	1 (14)	6 (86)	5.0
2	Is your dog fed a diet of nutritionally complete food, such as a high-quality commercial dry food? *	0 (0)	0 (0)	1 (14)	2 (29)	4 (57)	5.0
3	If your dog is fed a diet of raw food, are measures in place to avoid cross-contamination?	3 (43)	0 (0)	2 (29)	1 (14)	1 (14)	2.0
4	Is your dog fed a diet suitable for their age, size, and physical activity level?	0 (0)	0 (0)	2 (29)	1 (14)	4 (57)	4.5
5	Is your dog fed the right amount of food to maintain optimum weight?	1 (14)	1 (14)	0 (0)	2 (29)	3 (43)	4.0
6	Does your dog have a good feeding routine? E.g., is your dog fed at roughly the same time every day?	0 (0)	3 (43)	2 (29)	2 (29)	0 (0)	2.5
7	Is your dog fed at a suitable time each day? E.g., not immediately before exercising?	0 (0)	0 (0)	3 (43)	0 (0)	4 (57)	4.0
8	If recommended by your vet, does your dog receive supplements and vitamins as directed?	0 (0)	1 (14)	3 (43)	2 (29)	1 (14)	3.0
9	Is your dog given chews to aid in dental hygiene?	0 (0)	0 (0)	5 (71)	1 (14)	1 (14)	3.0
10	Is your dog’s place of living comfortable, protected from the weather, and not too hot or cold? *	0 (0)	0 (0)	1 (14)	2 (29)	4 (57)	4.5
11	Is your dog’s place of living safe? This includes a fully fenced yard or home with no holes that the dog could escape through, removing hazards, and ensuring safety from stressful situations, long periods of loud noise, and very high or low temperatures. *	0 (0)	0 (0)	1 (14)	1 (14)	5 (71)	5.0
12	If your dog is housed in high-density living such as a unit or apartment, are they regularly taken outside for exercise, play, and toileting? *	0 (0)	1 (14)	0 (0)	1 (14)	5 (71)	5.0
13	Does your dog have a space to retreat and withdraw to whenever they need downtime and rest? *	0 (0)	0 (0)	1 (14)	1 (14)	5 (71)	5.0
14	Is your dog given the ability to explore and become familiar with new environments?	0 (0)	0 (0)	2 (29)	2 (29)	3 (43)	4.0
15	Does your dog have outside space to enjoy, play, and explore, or does your dog receive plenty of time out and about with you?	0 (0)	0 (0)	2 (29)	1 (14)	4 (57)	4.5
16	If your dog is transported, such as in a car, are they safely restrained so as not to run around the vehicle or injure themselves?	0 (0)	0 (0)	2 (29)	1 (14)	4 (57)	4.5
17	If your dog is transported, do you take them out of the vehicle with you, rather than leaving them inside the vehicle unattended?	1 (14)	0 (0)	1 (14)	2 (29)	3 (43)	4.0
18	If your dog is going to be outside the home for a long period of time, have you ensured that they will have access to food, water, and comfortable bedding? *	0 (0)	1 (14)	0 (0)	1 (14)	5 (71)	5.0
19	Does your dog have a vet visit every 6–12 months? This should include a general check-up of teeth, eyes, respiratory, and circulatory system, and behaviour, as well as relevant worming, vaccinations, and tick prevention. *	0 (0)	0 (0)	0 (0)	1 (14)	6 (86)	5.0
20	Are you aware of what signs to look for that may indicate your dog is unwell, such as bloating, lethargy, and loss of appetite? *	0 (0)	1 (14)	0 (0)	1 (14)	5 (71)	5.0
21	Is your dog’s coat kept groomed to a high standard, including regular baths, grooming, and clipping as needed? *	0 (0)	0 (0)	0 (0)	4 (57)	3 (43)	4.0
22	Is your dog exercised at least once per day such as daily walks and/or play time? *	0 (0)	0 (0)	0 (0)	2 (29)	5 (71)	5.0
23	Is your dog on a worm, parasite, and tick prevention programme as recommended by your vet? *	0 (0)	0 (0)	1 (14)	3 (43)	3 (43)	4.0
24	Is your dog still fit to work? Do you notice signs associated with ageing and declining health that may inhibit their ability to work alongside you?	0 (0)	0 (0)	2 (29)	1 (14)	4 (57)	4.5
25	Are you aware of what constitutes normal behaviour and what constitutes unusual behaviour in a dog? *	0 (0)	1 (14)	0 (0)	1 (14)	5 (71)	5.0
26	Is your dog even-tempered, friendly, and sociable?	1 (14)	0 (0)	2 (29)	1 (14)	3 (43)	3.5
27	Does your dog show signs of unusual behaviour such as lip-licking, ears back, tail down, cowering, shivering, or outbursts of fear and aggression?	1 (14)	0 (0)	1 (14)	1 (14)	4 (57)	4.5
28	Is your dog willing to engage in play and exercise? *	0 (0)	0 (0)	1 (14)	3 (43)	3 (43)	4.0
29	Does your dog interact with humans and other animals in a manner that is sociable, friendly, and respectful? *	0 (0)	0 (0)	1 (14)	2 (29)	4 (57)	4.5
30	Does your dog have opportunities to socialise and interact with other dogs when they are not working? *	0 (0)	1 (14)	0 (0)	3 (43)	3 (42)	4.0
31	Do you ensure your dog has good experiences frequently? This may include playtime, socialisation with other dogs and humans, special treats, and positive affirmation. *	0 (0)	1 (14)	0 (0)	2 (29)	4 (57)	4.5
32	Does your dog seem to enjoy the work that they do? *	0 (0)	1 (14)	0 (0)	2 (29)	4 (57)	4.5
33	Does your dog seem to enjoy going on outings with you? *	0 (0)	1 (14)	0 (0)	2 (29)	4 (57)	4.5
34	Do you notice signs of anxiety or depression in your dog such as pacing, spinning, chasing shadows, chewing, or becoming withdrawn?	1 (14)	1 (14)	0 (0)	1 (14)	4 (57)	4.5
35	Do you allow your dog to live a balanced life, with enough downtime hours to offset working hours, or are they always on alert?	0 (0)	2 (29)	0 (0)	1 (14)	4 (57)	4.5
36	If your dog engages in strenuous activity or shows signs of fatigue, do they have enough downtime to recover?	0 (0)	2 (29)	0 (0)	2 (29)	3 (43)	4.0
37	If you notice fear and anxiety increasing in your dog, do you try to remove them from the situation that is causing the fear and anxiety? *	0 (0)	0 (0)	1 (14)	1 (14)	5 (71)	5.0
38	Do you ensure that your dog has the choice to engage or not engage in different activities? E.g., not forcing them to be petted or played with and allowing them the ability to withdraw from the situation at any time?	1 (14)	1 (14)	0 (0)	2 (29)	3 (43)	4.0

* Item reached a majority consensus (≥75%), based on the total of 4-point and 5-point responses as a proportion of all responses.

**Table 3:** Amendments to prototype items based on round two feedback.

**Round One Prototype Item**	**Amendment**	**Amendment Type**
Does your dog have a good feeding routine? E.g., is your dog fed at roughly the same time every day? Is your dog fed at a suitable time each day? E.g., not immediately before exercising?	Is your dog fed at roughly the same time each day and not immediately before vigorous exercise?	Merged due to feedback about similarity.
Is your dog fed a diet suitable for their age, size, and physical activity level? Is your dog fed the right amount of food to maintain optimum weight? Is your dog fed a diet of nutritionally complete food, such as a high-quality commercial dry food?	Is your dog fed a diet of nutritionally complete food, suitable for their size and physical activity level, as approved by a vet?	Merged due to feedback about similarity.
Does your dog have outside space to enjoy, play, and explore, or does your dog receive plenty of time out and about with you? Is your dog exercised at least once per day such as daily walks and/or playtime?”	Is your dog exercised at least once per day, such as daily walks and/or playtime outside?	Merged due to relevance.
Is your dog even-tempered, friendly, and sociable?		Omitted due to similarity to another item.
If your dog engages in strenuous activity or shows signs of fatigue, do they have enough downtime to recover?		Omitted due to similarity to another item.
If your dog is fed a diet of raw food, are measures in place to avoid cross-contamination?		Omitted due to low agreement (<50%).
If recommended by your vet, does your dog receive vitamins and supplements as directed?		Retained due to positive comments.
Is your dog given chews to aid in dental hygiene?		Retained due to positive comments.

**Table 4:** Round three items and agreement using a 5-point Likert scale (*n* = 6), where 1 = not at all important, 2 = somewhat not important, 3 = neutral, 4 = somewhat important, and 5 = extremely important. Note: All items reached a majority consensus (≥75%), based on the total of 4-point and 5-point responses as a proportion of all responses, except for items 3, 4, and 5.

Round Three Prototype Item		1-Point (%)	2-Point (%)	3-Point (%)	4-Point (%)	5-Point (%)	Mdn
1	Does your dog have access to fresh, clean water whenever they need it?	0 (0)	0 (0)	0 (0)	0 (0)	6 (100)	5.0
2	Is your dog fed a diet of nutritionally complete food, suitable for their size and physical activity level, as supported by a vet?	0 (0)	0 (0)	0 (0)	3 (50)	3 (50)	4.5
3	Is your dog fed at roughly the same time each day and not immediately before vigorous exercise?	0 (0)	0 (0)	2 (33)	1 (17)	3 (50)	4.5
4	If recommended by your vet, does your dog receive supplements and vitamins as directed?	0 (0)	1 (7)	3 (50)	2 (33)	0 (0)	3.0
5	Is your dog given chews to aid in dental hygiene?	0 (0)	0 (0)	4 (67)	1 (17)	1 (17)	3.0
6	Is your dog’s place of living comfortable, protected from the weather, and not too hot or cold?	0 (0)	0 (0)	0 (0)	0 (0)	6 (100)	5.0
7	Is your dog’s place of living safe? This includes a home with no holes that the dog could escape through, a fully fenced outdoor yard if applicable, removing hazards, and ensuring safety from stressful situations, long periods of loud noise, and very high or low temperatures.	0 (0)	0 (0)	0 (0)	0 (0)	6 (100)	5.0
8	If your dog lives indoors, are they regularly taken outside for exercise, play, and toileting?	0 (0)	0 (0)	1 (17)	1 (17)	4 (67)	5.0
9	Does your dog have a space to retreat and withdraw to whenever they need downtime and rest?	0 (0)	0 (0)	0 (0)	0 (0)	6 (100)	5.0
10	Is your dog given the ability to explore and become familiar with new environments?	0 (0)	0 (0)	0 (0)	2 (33)	4 (67)	5.0
11	If your dog is transported, such as in a car, are they safely restrained such as with a harness or crate, or trained by a professional trainer to sit in one position?	0 (0)	0 (0)	1 (17)	0 (0)	5 (83)	5.0
12	When inside a vehicle, do you ensure that it is a suitable temperature for the dog, not too hot or too cold?	0 (0)	1 (17)	0 (0)	1 (17)	4 (67)	5.0
13	If your dog is going to be outside the home for a long period of time, have you ensured that they will have access to food, water, and comfortable bedding?	0 (0)	0 (0)	1 (17)	1 (17)	4 (67)	5.0
14	According to the WSAVA Body Condition Score, does your dog have a score of 4 or 5?	0 (0)	0 (0)	0 (0)	4 (67)	2 (33)	4.0
15	Does your dog have a vet visit every 6–12 months? This should include a general check-up of teeth, eyes, respiratory and circulatory system, weight and behaviour, as well as relevant worming, vaccinations, and tick prevention.	0 (0)	0 (0)	0 (0)	1 (17)	5 (83)	5.0
16	Are you aware of what signs to look for that may indicate your dog is unwell, such as bloating, lethargy, and loss of appetite?	0 (0)	0 (0)	1 (17)	1 (17)	4 (67)	5.0
17	Is your dog’s coat kept groomed to a high standard, including regular baths, grooming, and clipping as needed?	0 (0)	0 (0)	0 (0)	5 (83)	1 (17)	4.0
18	Is your dog exercised at least once per day such as daily walks and/or play time?	0 (0)	0 (0)	0 (0)	1 (17)	5 (83)	5.0
19	Is your dog on a worm, parasite, and tick prevention programme as recommended by your vet?	0 (0)	0 (0)	1 (17)	2 (33)	3 (50)	4.5
20	Do you notice signs associated with ageing and declining health that may inhibit your dog’s ability to work alongside you?	0 (0)	0 (0)	1 (17)	1 (17)	4 (67)	5.0
21	Are you aware of what constitutes normal, content behaviour and what constitutes negative behaviour in a dog?	0 (0)	1 (17)	0 (0)	2 (33)	3 (50)	4.5
22	Does your dog show signs of behaviour such as lip-licking, ears back, tail down, cowering, shivering, or outbursts of fear and aggression?	0 (0)	0 (0)	1 (17)	0 (0)	5 (83)	5.0
23	Is your dog willing to engage in play and exercise?	0 (0)	0 (0)	0 (0)	2 (33)	4 (67)	5.0
24	Does your dog interact with humans and other animals in a manner that is sociable, friendly, and respectful?	0 (0)	0 (0)	0 (0)	1 (17)	5 (83)	5.0
25	Do you ensure your dog has good experiences frequently? This may include playtime, socialisation with other dogs and humans, and special treats.	0 (0)	0 (0)	0 (0)	1 (17)	5 (83)	5.0
26	Does your dog seem to enjoy the work that they do? Signs may include tail wagging, interest in their work/task, or willingness to participate.	0 (0)	0 (0)	0 (0)	2 (33)	4 (67)	5.0
27	Does your dog seem to enjoy going on outings with you? Signs may include tail wagging, sniffing and exploring the environment, or willingness to go out.	0 (0)	0 (0)	0 (0)	2 (33)	4 (67)	5.0
28	Do you think that your dog has a good work–life balance, with enough downtime to offset working hours?	0 (0)	0 (0)	0 (0)	2 (33)	4 (67)	5.0
29	Are you aware of behaviours that may indicate anxiety and depression in a dog?	0 (0)	0 (0)	0 (0)	2 (33)	4 (67)	5.0
30	If you notice fear and anxiety increasing in your dog, do you try to remove them from the situation that is causing the fear and anxiety?	0 (0)	0 (0)	0 (0)	1 (17)	5 (83)	5.0
31	Do you ensure that your dog has the choice to engage or not engage in different activities if they become too much? E.g., not forcing them to be petted or played with and allowing them the ability to withdraw from the situation at any time?	0 (0)	0 (0)	0 (0)	1 (17)	5 (83)	5.0

**Table 5:** Final changes to prototype items based on round three feedback.

**Round Three Prototype Item**	**Final Decision**
If recommended by your vet, does your dog receive supplements and vitamins as directed?	Omitted due to low agreement (<50%).
Is your dog given chews to aid in dental hygiene?	Omitted due to low agreement (<50%).
Is your dog fed at roughly the same time each day and not immediately before vigorous exercise?	Retained as an optional item, open-ended feedback indicated that it may be best to split the question into two parts.
Is your dog on a worm, parasite, and tick prevention programme as recommended by your vet?	Omitted due to similarity to another item.

**Table 6:** User responses to HERO tool (*n* = frequency of responses by 23 participants).

Item		Yes	No	Unsure
1	Does your dog have access to fresh, clean water whenever they need it?	23	0	0
2	Is your dog fed a diet of nutritionally complete food, suitable for their size and physical activity level, as supported by a vet?	21	1	1
3	Is your dog’s place of living comfortable, protected from the weather, and not too hot or cold?	23	0	0
4	Is your dog’s place of living safe? This includes an escape-proof home and yard with no hazards.	23	0	0
5	If your dog lives indoors, are they regularly taken outside for exercise, play, and toileting?	23	0	0
6	Does your dog have a space to retreat and withdraw to whenever they need downtime and rest?	23	0	0
7	Is your dog given the ability to explore and become familiar with new environments?	22	1	0
8	If your dog is transported, such as in a car, are they safely restrained such as with a harness or crate, or trained by a professional trainer to sit in one position?	23	0	0
9	When inside a vehicle, do you ensure that it is a suitable temperature for the dog, not too hot or too cold?	23	0	0
10	If your dog is going to be outside the home for a long period of time, have you ensured that they will have access to food, water, and comfortable bedding?	23	0	0
11	(Show the WSAVA Body Condition Graphic). According to the WSAVA Body Condition Score, does your dog have a score of 4 or 5?	21	1	1
12	Does your dog have a vet visit every 6–12 months? This should include a general check-up of teeth, eyes, respiratory and circulatory system, weight and behaviour, as well as relevant worming, vaccinations, and tick prevention.	23	0	0
13	Are you aware of what signs to look for that may indicate your dog is unwell, such as bloating, lethargy, and loss of appetite?	23	0	0
14	Is your dog’s coat kept groomed to a high standard, including regular baths, grooming, and clipping as needed?	21	0	2
15	Is your dog exercised at least once per day, such as daily walks and/or playtime outside?	22	1	0
16	Do you know what signs to look for that may indicate ageing or declining health which could inhibit your dog’s ability to work alongside you?	21	0	2
17	Are you aware of what constitutes positive behaviour and what constitutes negative behaviour in a dog?	23	0	0
18	Do you notice behaviours such as lip-licking, ears back, tail down, cowering, shivering, snarling, or aggression in your dog regularly?	7	16	0
19	Is your dog willing to engage in play and exercise?	22	1	0
20	Does your dog interact with humans and other animals in a manner that is sociable, friendly, and respectful?	22	1	0
21	Do you ensure that your dog has good experiences frequently? This may include playtime, socialisation with other dogs and/or humans, and receiving special treats.	23	0	0
22	Does your dog seem to enjoy the work that they do? Signs may include tail wagging, interest in their work/task, or willingness to participate.	23	0	0
23	Does your dog seem to enjoy going on outings with you? Signs may include tail wagging, sniffing, and exploring the environment or willingness to go out.	23	0	0
24	Do you think that your dog has a good work–life balance, with enough downtime to offset working hours?	23	0	0
25	Are you aware of behaviours that may indicate anxiety and depression in a dog?	20	2	1
26	If you notice fear and anxiety increasing in your dog, do you try to remove them from the situation that is causing the fear and anxiety?	21	1	1
27	Do you ensure that your dog has the choice to engage or not engage in activities if they become too much? E.g., never forcing them to be petted, played with, or engage in work.	23	0	0
28	Optional—Is your dog fed at roughly the same time each day and not immediately before vigorous exercise?	23	0	0

**Table 7:** User ratings on HERO usability and usefulness (*n* = 23). Participant responses were based on a 5-point Likert scale with ‘5’ indicating ‘strongly agree’ and ‘1’ indicating ‘strongly disagree’.

Statement	M (SD)	Mdn
The assessment tool was easy to understand and straightforward.	4.8 (0.4)	5.00
The assessment tool was quick to use.	4.7 (0.4)	5.00
The wording was clear to read and understand.	4.9 (0.3)	5.00
The pictures were clear to see and understand.	4.5 (0.8)	5.00
The additional resources were informative and easily accessed.	4.4 (0.8)	5.00
The assessment tool is valuable in helping you understand what your dog needs.	4.5 (0.7)	5.00
The assessment tool is valuable in helping you assess which areas of your dog’s lifestyle can be improved.	4.3 (0.8)	5.00

**Table 8:** Thematic analysis of usefulness and usability open-ended feedback (*n* = 23).

Question	Theme (*n*)	Supporting Quote
Did you encounter any difficulties in using the survey? Please indicate the difficulties faced.	Ease of use and lack of difficulties (15)	“No” “No difficulties”
	Availability of information from training centres (1)	“No difficulties but training centre provides us with this info and conducts upskilling sessions. So, nothing new here for me”
	Purpose of the survey (2)	“Had no difficulty in taking this survey. But to me it is a SURVEY and not a teaching tool.” “No difficulties but training centre provides us with this info and conducts upskilling sessions. So, nothing new here for me”
	Simplicity and straightforwardness (2)	“No, it was easy and straightforward” “No—it was basic and easy.”
	Need for more options or applicability (2)	“In some areas might need more options” “No not really although yes/no answers don’t always apply. Not applicable in some answers”
	Positive engagement and enjoyment (1)	“I enjoyed sharing my experiences with my service dog, Joy. Very good survey”
	Suggestion for inclusion of Auslan (1)	“In Auslan will be good”
Were there any parts of the survey that you enjoyed or found useful? Please share this with us.	Interest and relevance of information (7)	“All the side information/websites were very interesting and informative”“The survey was very interesting and informative, thank you.” “Found very useful.” “The health care and medical needs of the dogs.”
	Reinforcement of existing knowledge (2)	“They were things I already know. We had dogs growing up and prior to me receiving my first hearing dog. So knew a lot of this information already. But it was good to enforce what I already know.”
	Intent to explore further information (1)	“I will certainly read through some of the linked information”
	No change in behaviour (2)	“Nothing in the survey has made me think twice about anything. Also, I would not change anything I do after participating in this survey.”
	Common sense questions and relevance (1)	“I thought the questions were just common-sense dog owner questions and didn’t really relate in any way to hearing dogs alone.”
	Positive impact of Hearing Dogs (1)	“My dog has made such a difference to my life. I feel very safe and secure with him. He is trained to work the smoke alarm, and I am 100% confident that in that event he would alert me if alarm sounded.
	Routine testing and training (1)	“At night I can’t hear anything because my hearing aids are removed. He is trained to jump on the bed, touch me and then drop to the floor when I sit up. My husband and I test him with smoke alarm every week. His other sounds happen daily which he always works. He barks when doorbell or knock happens. He shouldn’t but I don’t consider it bad behaviour because if this happened at night, I would then know the wrong person is hanging around.”
	Neutral or no response (4)	“No”
Do you have any other feedback or suggestions pertaining to the survey?	Access to information and websites (1)	“How can I access all the information/websites as a ‘bundle’ for future reference, and to share with family/friends (NOT by FB or Twitter!)”
	Lack of additional feedback (8)	“No”
	Uncertainty about the survey audience (1)	“I am not sure who this would be directed to when you consider the training we are given to care for our dogs”
	Interest and importance of topics (2)	“I do feel some people need remind about the heat and the ground and offer suggestions for them. and snake kit is very important.” “I won’t waste 10 min on it in future!”
	Interest in comparing responses (1)	“It would be very interesting to know how other hearing assistance dog owners responded to this survey.”
	Challenges with public access and airlines (1)	“Virgin is the only airline that seems to welcome hearing dogs on flights. I feel the message needs to get out to other airlines that these dogs should be able to fly without hassle with their recipients.”
	Pride in Hearing Dogs (1)	“You truly need to see him in action as he is so precious, and I am so proud of him and his behaviour. I am very fortunate to have him. I am 75 and we grow old together.”
	Availability of dog trainer (1)	“I also have a dog trainer I can ask questions about”
	Unique needs of Hearing Dogs (1)	“Our dogs are unique. For instance, the question about leaving dog at home with food and water does not apply. Would never leave my dog”

## Data Availability

Full data available from the authors upon request.
